# Activation and regulation mechanisms of NOD-like receptors based on structural biology

**DOI:** 10.3389/fimmu.2022.953530

**Published:** 2022-09-15

**Authors:** Umeharu Ohto

**Affiliations:** Graduate School of Pharmaceutical Sciences, University of Tokyo, Tokyo, Japan

**Keywords:** innate immunity, pathogen-associated molecular patterns, damage-associated molecular patterns, pattern recognition receptors, NOD-like receptors (NLRs), inflammasome

## Abstract

Innate immunity is a primary defense system against microbial infections. Innate immune pattern recognition receptors (PRRs) play pivotal roles in detection of invading pathogens. When pathogens, such as bacteria and viruses, invade our bodies, their components are recognized by PRRs as pathogen-associated molecular patterns (PAMPs), activating the innate immune system. Cellular components such as DNA and RNA, acting as damage-associated molecular patterns (DAMPs), also activate innate immunity through PRRs under certain conditions. Activation of PRRs triggers inflammatory responses, interferon-mediated antiviral responses, and the activation of acquired immunity. Research on innate immune receptors is progressing rapidly. A variety of these receptors has been identified, and their regulatory mechanisms have been elucidated. Nucleotide-binding and oligomerization domain (NOD)-like receptors (NLRs) constitute a major family of intracellular PRRs and are involved in not only combating pathogen invasion but also maintaining normal homeostasis. Some NLRs are known to form multi-protein complexes called inflammasomes, a process that ultimately leads to the production of inflammatory cytokines and induces pyroptosis through the proteolytic cascade. The aberrant activation of NLRs has been found to be associated with autoimmune diseases. Therefore, NLRs are considered targets for drug discovery, such as for antiviral drugs, immunostimulants, antiallergic drugs, and autoimmune disease drugs. This review summarizes our recent understanding of the activation and regulation mechanisms of NLRs, with a particular focus on their structural biology. These include NOD2, neuronal apoptosis inhibitory protein (NAIP)/NLRC4, NLR family pyrin domain containing 1 (NLRP1), NLRP3, NLRP6, and NLRP9. NLRs are involved in a variety of diseases, and their detailed activation mechanisms based on structural biology can aid in developing therapeutic agents in the future.

## Introduction

Bacteria and viruses invading our bodies are recognized as foreign substrates and, therefore, activate the immune system. Immune responses are classified into innate and acquired immunity. In the early stages of microbial invasion, innate immunity is triggered first (1, 2). Pathogen-associated molecular patterns (PAMPs) are recognized by an innate immune receptor called pattern recognition receptor (PRR) (3). Signals are transmitted downstream, ultimately triggering inflammatory responses, interferon-mediated antiviral responses, and the activation of acquired immunity (4). The discovery of toll-like receptors (TLRs) in the late 1990s led to an explosion of research on innate immune receptors, resulting in the identification of a variety of innate immune receptors (5). Each of these receptors was found to be involved in the recognition of unique PAMPs (6). Previously, innate immunity was considered a nonspecific immune response; however, it has now been recognized as a specific immune response due to PRRs (7). In addition to recognizing PAMPs, innate immune receptors may be activated by self-derived molecular patterns (damage-associated molecular patterns, DAMPs) released from necrotic cells, which are known to cause autoimmune diseases (8). Therefore, innate immune receptors are potential drug targets, such as for antiviral drugs, immunostimulants, antiallergic drugs, and drugs for autoimmune diseases (9).

In addition to the aforementioned TLRs, representative innate immune receptors have been identified as nucleotide-binding and oligomerization domain (NOD)-like receptors (NLRs) (10), retinoic acid-inducible gene I (RIG I)-like receptors (11), absent in melanoma 2 (AIM2)-like receptors (12), and cyclic GMP-AMP synthase (cGAS)/stimulator of interferon genes (STING) (13). TLRs are located on the plasma membrane surface and endosomal membranes, whereas other receptors are located in the cytoplasm. Moreover, TLRs recognize PAMPs and DAMPs that have entered the cell. Recently, a rapid progress in the study of intracellular sensors that exist in the cytoplasm and activate innate immunity by recognizing foreign substances, such as pathogen-derived DNA and RNA, has been observed (14, 15). Structural biology studies using X-ray crystallography and cryo-electron microscopy (EM) have made remarkable progress in recent years. Moreover, these studies have played a major role in elucidating the mechanisms by which innate immune receptors recognize PAMPs and DAMPs and activate innate immunity. This review focuses on NLRs, a family of innate immune receptors that exist primarily in the cytoplasm, and introduces the mechanisms of activity regulation and signal transduction revealed by structural biology studies conducted over the past decade (**Table 1**).

**Table 1:** Summary of structural studies of NLRs.

	Structural features
NLRC4	Inactive form (monomer) (16) Active form (ring-like oligomer complexed with NAIP/ligand) (17–21)
NLRP3	Inactive form (NEK7-bound monomer) (22) Inactive form (cage-like oligomer) (23–25) Inhibitor bound form (23, 24, 26)
NLRP1	Inactive C-terminal fragment (DPP9-bound form) (27, 28)
NOD2	Inactive form (monomer) (29)
NLRP9	Inactive form (monomer) (30)

### NOD-like receptors

**Figure 1:**
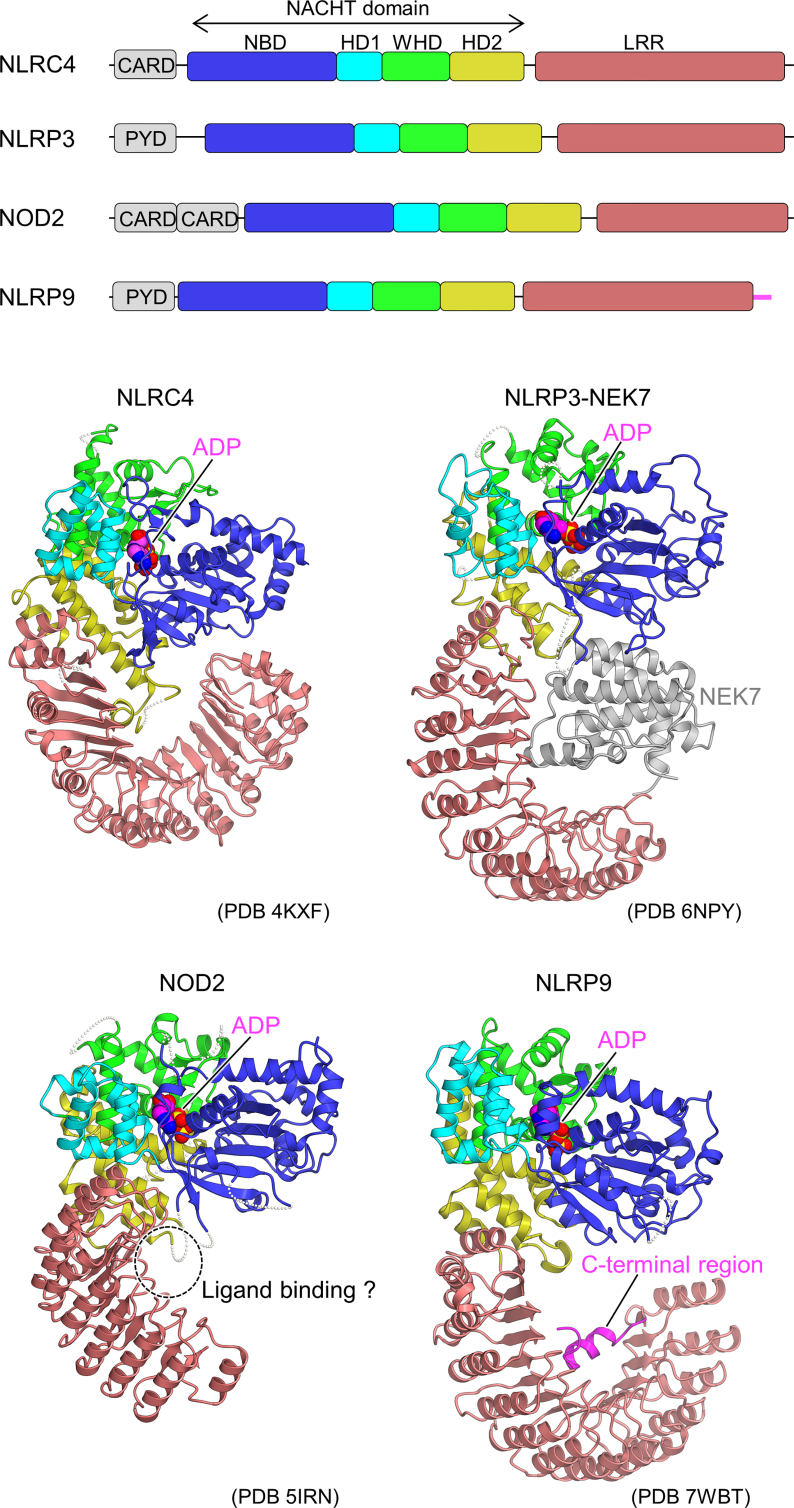
Domain organization and structures of inactive NLRs. **(A)** Domain organization of NLRs. Each of the domains and sub domains are indicated by the different colors and are correspondingly indicated in Figure 1B. **(B)**, Structures of inactive NLRs. Structures of inactive NLRC4 (PDB 4KXF) (16), NLRP3-NEK7 complex (PDB 6NPY) (36), NOD2 (PDB 5IRN) (29), and NLRP9 (PDB 7WBT) (30) are shown with the domains colored as per **(A)**. Bound ADP molecules are shown in space filling representations. In the NLRP3-NEK7 complex, bound NEK7 is shown in gray. The potential ligand-binding site in NOD2 and the C-terminal region of NLRP9 are indicated. Structural figures were generated using CueMol throughout this review (http://www.cuemol.org).

To date, 22 NLRs with various functional roles have been identified in humans (31). NLR typically consists of three functional domains, namely N-terminal signaling, central [NAIP, CIITA, HETE, TP1 (NACHT)], and leucine-rich repeat (LRR) domains (32–35) (**Figure 1A**). The N-terminal signaling domain is responsible for signal transduction through interactions with downstream adaptor proteins. The central NACHT domain has ATPase activity and is assumed to be self-oligomerized through this domain on activation. The NACHT domain is further classified into nucleotide-binding domain (NBD), helical domain 1 (HD1), winged-helix domain (WHD), and HD2 subdomains. The LRR domain on the C-terminal side is believed to be involved in ligand recognition and functional regulation. NLRs are classified into subfamilies according to the type of N-terminal signaling domain: those with pyrin domain (PYD) are called the NLR pyrin domain containing (NLRP) family and those with caspase recruitment domain (CARD) are called the NLR CARD containing (NLRC) family. An NLRP uses its PYD to form a scaffold that interacts with the adaptor, apoptosis-associated, speck-like protein containing a CARD (ASC) through PYD-PYD interactions to recruit procaspase-1. Procaspase-1 is activated to caspase-1, which further cleaves pro-interleukin (IL)-1β and pro-IL-18, resulting in the generation of mature IL-1β and IL-18, respectively, and triggering an inflammatory response. Caspase-1 also induces pyroptosis by cleaving gasdermin D. In addition to the caspase-1-mediated canonical inflammasomes, noncanonical inflammasomes involving caspase-4/5 in human and caspase-11 in mice have been identified and are known to respond to cytosolic LPS (37). An NLRC, however, is thought to activate caspase-1 through direct CARD-CARD interactions in addition to the ASC-mediated activation of caspase-1. Diverse PAMPs and DAMPs have been found to activate NLRs. Some NLRs are known to activate innate immunity by forming high-molecular-weight multi-protein complexes called inflammasomes to signal downstream.

### NLRC4

In the neuronal apoptosis inhibitory protein (NAIP)/NLRC4 pathway, flagellin, a component of bacterial flagella, and the bacterial rod protein PrgJ bind to NAIPs, whereupon NLRC4 binds as an adapter to form active inflammasome (38–43).

In 2013, the first crystal structure of NLR was reported for the inactive form of NLRC4, lacking the CARD domain (16) (**Figure 1B**). The inactivated conformation of NLRC4 was a monomeric, autoinhibited conformation, in which the region of the NACHT domain involved in self-association was covered by the LRR domain. Thus, the LRR domain of NLRC4 functions to sequester NLRC4 in a monomeric state. It was also found that ADP bound to the NACHT domain stabilizes the closed and autoinhibited conformation of NLRC4 by mediating the interactions between the subdomains of the NACHT domain. This was consistent with previous biochemical experiments showing that the deletion of the LRR domain leads to self-activation without NAIP or FliC (39).

**Figure 2:**
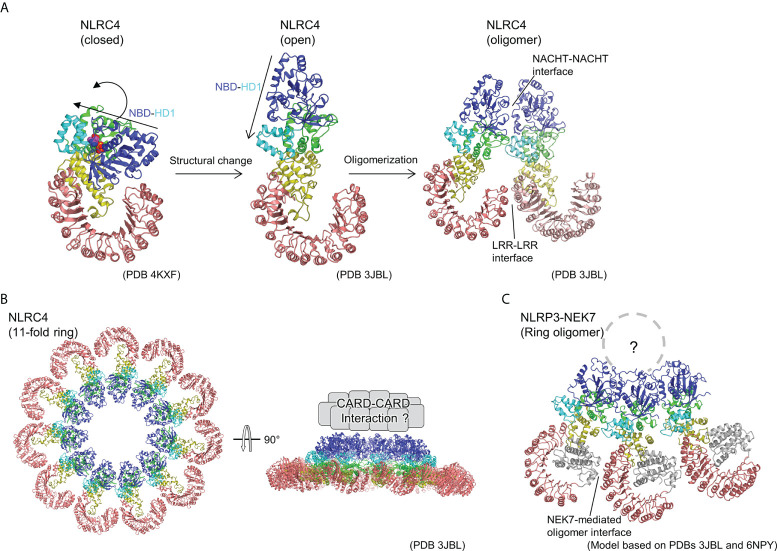
Ring-shaped active oligomer of NLRC4. **(A)** Structural changes underlying NLRC4 oligomerization. The NLRC4 monomer undergoes a structural change from a closed (PDB 4KXF) (16) to an open form (PDB 3JBL) (17), causing the NBD-HD1 part to undergo a large rotational movement relative to the other parts. This opens the NACHT domain and the corresponding activated NLRC4 molecules to form a laterally aligned dimer and subsequently form ring-shaped oligomer. **(B)** Structure of 11-fold ring-shaped NLRC4 oligomer (PDB 3JBL) (17). Top (left) and side (right) views are shown. The CARD domains are predicted to be concentrated at the top of the ring as shown schematically in the side view (right). **(C)** Hypothetical structure of NLR3-NEK7 oligomer. The structure of the inactive form of the NLRP3-NEK7 complex (6NPY) (36) was split into the NBD-HD1 and the WHD-HD2-LRR parts, and each was fitted into the corresponding part of the 11-fold ring oligomer of NLRC4 (PDB 3JBL) (17).

Subsequently, cryo-EM analysis revealed that the inflammasome structure of NAIP2-NLRC4 is induced by the bacterial rod protein PrgJ, as reported almost simultaneously by two groups (17, 18) (**Figure 2**). A low-resolution structure of the NAIP5-NLRC4 helical oligomer induced by flagellin was also reported using cryo-EM tomography (19). Afterwards, cryo-EM structures of the flagellin-NAIP5-NLRC4 were reported, revealing the detailed ligand recognition mechanism of NAIP5 as well as how it leads to the oligomerization of NLRC4 (20, 21). The NAIP2-NLRC4 or NAIP5-NLRC4 oligomer induced by ligand forms a ring-like structure consisting of 10–12 molecules, including one NAIP molecule (**Figure 2B**). This oligomer is formed by unidirectional chain oligomerization of NLRC4 molecules, starting with the ligand-bound NAIP molecule. In the upper part of the ring, CARD assembly may provide a scaffold for CARD-CARD interactions with downstream caspase-1 (**Figure 2B**). Upon activation, closed NLRC4 is converted to an open conformation by binding to open NLRC4 *via* the NACHT domain (**Figure 2A**). During this process, rigid body motion at the linkage between HD1 and WHD of the NACHT domain is observed. This mechanism amplifies the signal by catalytically converting the closed structure to an open structure in a self-propagating manner. Moreover, this mechanism contrasts with the activation mechanism of the apoptotic protease-activating factor-1 (Apaf-1) apoptosome (octameric ring structure), which is related to the NLR. In the case of Apaf-1, each subunit must be activated by its own ligand, that is, a stoichiometric number of ligands is required for all subunits activation (44).

### NLRP3

NLRP3 is one of the most well-studied inflammasome-forming NLRs. Moreover, its activators are diverse. For instance, nigericin, uric acid, amyloid-beta fibrils, extracellular ATP, and reactive oxygen species are a few activators of NLRP3 (20, 35). Some of these are thought to trigger NLRP3 activation by lowering intracellular K^+^ concentrations (45). NLRP3 activation requires two steps of stimulation: “priming” and “activation” (45–47). Priming stimuli include the TLR ligands Pam3CSK4, Poly(I:C), lipopolysaccharide (LPS), and R848, which activate TLRs to upregulate NLRP3 and caspase-1 expression and provide the soil for NLRP3 activation. In addition, priming causes post-translational modifications in NLRP3, such as phosphorylation (48), which are thought to be important for NLRP3 activation. The activating factors include nigericin, extracellular ATP, as well as silica, cholesterol, and uric acid crystals that destabilize lysosomes. As mentioned previously, a wide variety of NLRP3 activators exists, and the direct triggers of NLRP3 activation remain unclear. In addition, it has been reported that NLRP3 activation involves interactions with a variety of proteins. These include SGT1, HSP90 (49), thioredoxin-interacting protein (TXNIP) (50), mitochondrial antiviral-signaling protein (MAVS) (51), never in mitosis A-related kinase 7 (NEK7) (52–54), MAP/microtubule affinity-regulating kinase 4 (MARK4) (55), macrophage migration inhibitory factor (MIF) (56), DEAD box RNA helicase (DDX) 3X (57), and receptor of activated protein C kinase 1 (RACK1) (58). However, the mechanisms by which these factors regulate NLRP3 activation remain unclear.

### NLRP3–NEK7 complex

As a starting point for the structural biology studies of NLRP3, the cryo-EM structure of inactivated human NLRP3 (PYD domain deleted) bound to NEK7 was first revealed (36) (**Figure 1B**). The overall structure was similar to the previously reported structures of NLRC4 (16) and NOD2 (29) in the inactivated form. The kinase C-lobe of NEK7 binds to the concave side of the LRR of NLRP3. Only the C-lobe of NEK7 was visible in the cryo-EM map, but the N-lobe did not clash with NLRP3 even when full-length NEK7 was superimposed. NLRP3 binds to NEK7 at multiple interaction sites (LRR, HD2, and NBD). This binding is suggested to involve electrostatic interactions between the positively charged surface of NEK7 and the negatively charged surface of NLRP3. NEK7 is known to form a complex with NEK9 to participate in mitosis (59); however, the NEK7 surface used for this complexation overlaps in part with the surface used for binding to NLRP3. Therefore, it was expected that once NEK7 binds to NLRP3, it cannot bind to NEK9 and vice versa.

As mentioned previously, upon activation, NLRC4 multimerizes and activates by opening the NACHT domain *via* a large rigid body rotation between HD1 and WHD (**Figure 2A**) (17, 18). Imitating the oligomeric structure of NLRC4, an oligomeric model of the NLRP3-NEK7 complex was constructed (**Figure 2C**), where NEK7 was found to be located at the boundary with the neighboring molecule in the oligomer. To investigate the importance of this modeled oligomeric interface, the authors of this paper performed experiments using mutants of NLRP3 and NEK7 and demonstrated that both mutants reduce NLRP3 activation, indicating that this NEK7–NLRP3 interface may be used when NLRP3 is activated (36). In the case of NLRC4, in addition to the contacts at the NACHT site, interactions at the LRR-LRR sites are observed during the formation of ring-shaped oligomers (**Figure 2A, B**) (17, 18). However, the LRR-LRR interaction in the NLRP3 oligomer is not possible between adjacent monomers because the LRR of NLRP3 is smaller than that of NLRC4. Considering the result of the mutational experiment showing the importance of hypothetical NEK7-NLRP3 interface describe earlier, NEK7-mediated bridging of adjacent LRRs of NLRP3 may reinforce the oligomerization of NLRP3. In other words, in NLRP3, as in the case of NLRC4, the interaction between NACHTs in the inner ring layer and that between LRRs *via* NEK7 in the outer ring layer are thought to contribute to oligomer formation.

### Full-length NLRP3 oligomer

**Figure 3:**
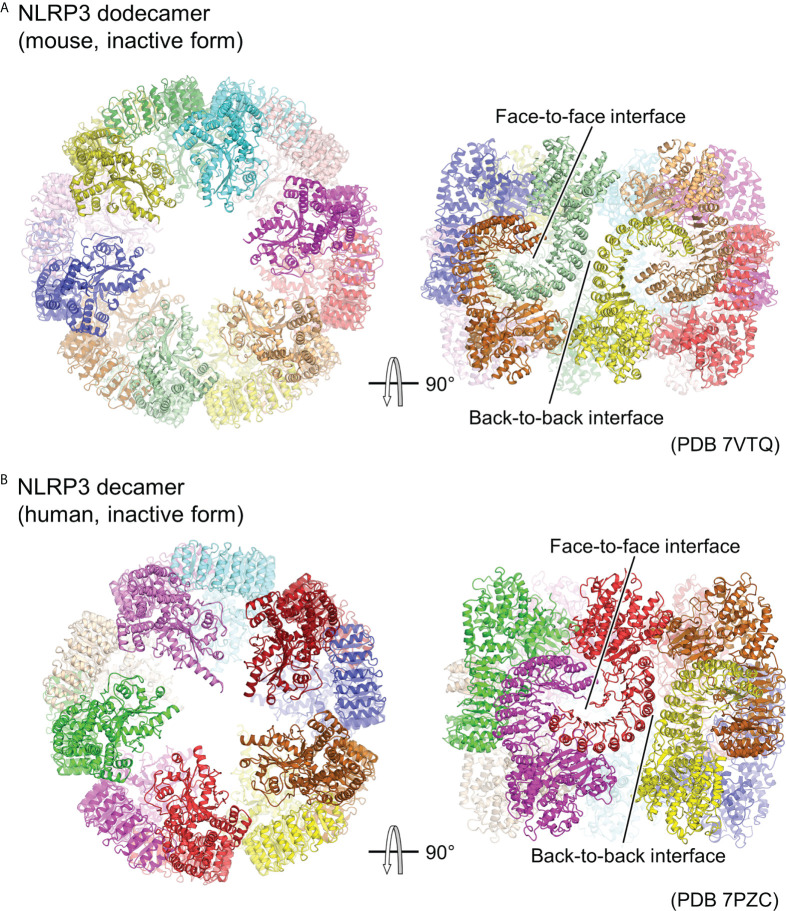
Cage-shaped inactive oligomer of NLRP3. Top and side views of the structure of **(A)** full-length mouse NLRP3 dodecamer (PDB 7VTQ) (23) and **(B)** Human NLRP3 decamer (PDB 7PZC) (24). Each protomer is shown in a different color. The LRR-mediated oligomer interfaces are indicated.

Although experimental structural information on the activated oligomer of NLRP3 is not yet available, three groups have reported cryo-EM structures of the full-length NLRP3 oligomer in its inactivated form recently (**Figure 3**) (23–25). Paradoxically, this inactivated oligomer formation has been shown to be important in the regulation of NLRP3 activation (25). Mouse NLRP3 forms 12-, 14-, and 16-mer (**Figure 3A**) (23, 25), whereas human NLRP3 forms 10-mer (**Figure 3B**) (24) hollow, cage-like oligomeric structures with NACHT on the top surface and LRR-LRR interactions on the sides. The density of the PYD domains could not be clearly confirmed, but they were considered to be disordered and located inside or at the top of the cage.

LRR-LRR interactions on the side of the cage are the main contributors to the multimer formation. The interactions at this site are “face-to-face” or “head-to-head,” in which neighboring LRRs interact closely with each other (**Figure 3**). These interactions are mainly due to electrostatic complementarity and hydrophobicity, respectively. In contrast, the NACHTs on the upper and lower surfaces of the cage are proximal to each other, but there is little direct interaction between them. As a result, in all the reported oligomer structures, the density of the LRR portions on the sides of the cage is clear, whereas that of the NACHT portions on the top and bottom surfaces of the cage is relatively obscure.

The structure of the NLRP3 protomer in the oligomer matches well with the previously reported structure of NLRP3 in the inactivated NLRP3-NEK7 structure (36). The LRR-LRR and NLRP3-NEK7 interaction interfaces overlap, suggesting that this cage-like NLRP3 oligomer cannot accommodate NEK7. Moreover, this suggests that the cage-like NLRP3 oligomer is reorganized when NEK7 binds to and activates NLRP3. Furthermore, it has been shown that adding NEK7 to the NLRP3 oligomer partially dissociates the oligomer (36). NEK7 is a centrosomal kinase that mainly localizes to the microtubule-organizing center (60, 61), where NLRP3 does not encounter NEK7 in resting cells, suggesting that spatial isolation is one of the mechanisms preventing NLRP3 from being unintentionally activated (25).

Although the density of PYD was not clearly identified in the cage-like oligomeric structure, it is likely that PYD contributes to the formation of this cage-like oligomer, as it does not form when PYD is deleted (23, 25). Moreover, the PYD-deleted form of human NLRP3 forms a hexamer, while intact human NLRP3 forms a cage-like decamer (29, 59). The cage-like NLRP3 oligomers did not induce downstream ASC filament formation (25), suggesting that the PYDs in the oligomers were confined or spatially constrained within the cage, thereby inhibiting filament formation (23–25). This has been proposed as one of the mechanisms limiting NLRP3 activation.

The cage-like NLRP3 oligomer has been shown to have an affinity for membranes (23, 25). Oligomers of NLRP3 have been detected in membrane-extracted fractions from HEK293T cells overexpressing NLRP3 or from immortalized bone marrow-derived macrophages that express NLRP3 endogenously by LPS stimulation (25). Lipid strip assay results have shown that NLRP3 has an affinity for acidic lipids such as phosphatidylinositides, phosphatidic acid, phosphatidyl serine, and cardiolipin (23, 25). This corresponds well with the localization of NLRP3 to acidic lipids in the trans-Golgi network (TGN) (62). Furthermore, thorough functional assay results indicated that the cage-like NLRP3 oligomer is essential for TGN dispersion and NLRP3 activation (25).

In summary, the following mechanism has been proposed (23–25): NLRP3 is localized as a cage-like oligomer on the TGN and mitochondrial membranes in the resting state, where its activation is inhibited by the confinement or structural restriction of PYD. NLRP3 is then activated by activation signals such as due to nigericin, which induces a conformational change to form an activated oligomer.

### NLRP3 inhibitor

**Figure 4:**
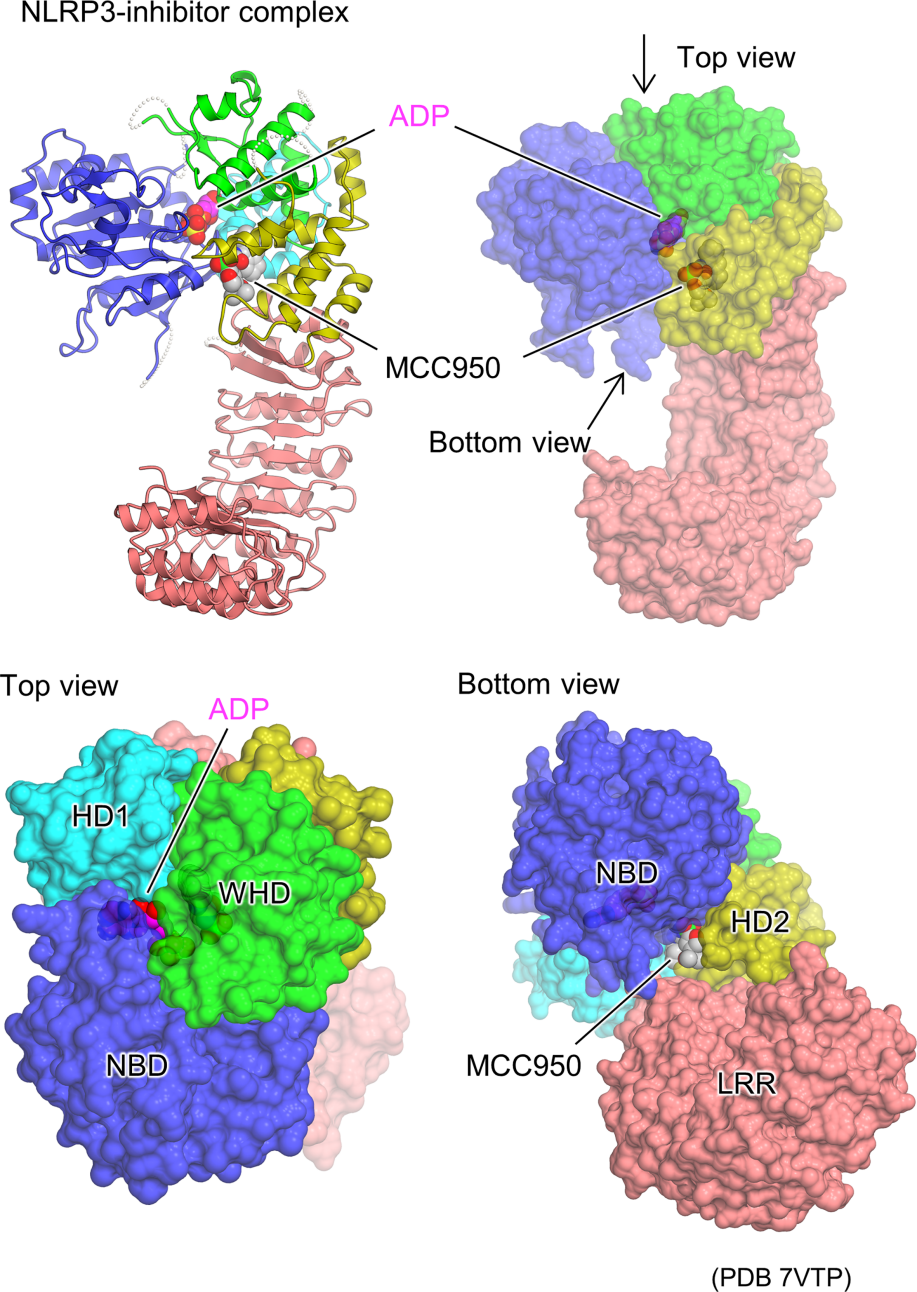
Structural basis of NLRP3 inhibitor binding. Protomer structure of the human NLRP3 (PYD deleted) hexamer (PDB 7VTP) (23) with bound molecules of the ADP and NLRP3 inhibitor, MCC950, is illustrated as space filling representations. Ribbon representation (top left) and surface representations from three different views (top right, bottom left, and bottom right) are demonstrated.

The cryo-EM structures of the artificial hexamer of human NLRP3 (PYD-deficient), full-length mouse NLRP3 dodecamer (23), and full-length human NLRP3 decamer (24) as well as the crystal structure of the NACHT domain of human NLRP3 (26) have been determined in the presence of the NLRP3 inhibitor MCC950 or its analogs (63–66). This revealed the inhibitor binding mode and the mechanism of inhibition of NLRP3 activation (**Figure 4**). The inhibitor binds to the bottom of the cavity in the NACHT domain. This cavity is composed of all the domains and subdomains of NLRP3. Although the inhibitor binds spatially close to the ADP binding site, the binding sites are separated by an interaction between NBD, HD1, and WHD, allowing the inhibitor to access NLRP3 from the NBD-HD2-LRR side, whereas ADP accesses NLRP3 from the opposite side. The closed conformation of NACHT domains is generally characterized by tight packing between NACHT subdomains *via* ADP binding (16, 29, 30, 36). Like ADP, the inhibitor binds to NLRP3 and mediates its interaction with its subdomain as well as with LRR. This suggests that inhibitors stabilize the closed conformation of the NACHT domain of NLRP3, thereby preventing the NACHT domain from changing to an open conformation and being activated (23, 24, 26).

### NLRP1

**Figure 5:**
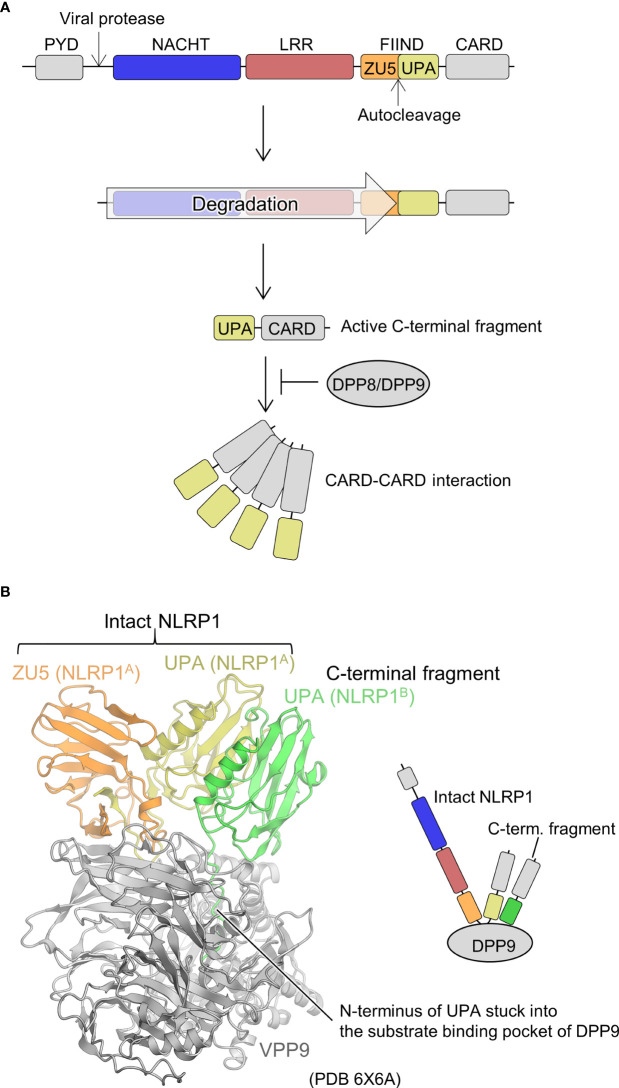
Mechanism underlying NLRP1 activation and DPP9-mediated suppression of NLRP1 activation. **(A)** NLRP1 activation mechanism. The domains are indicated in various colors and are correspondingly represented in Figure 5B. **(B)** Structure of the DPP9-NLRP1 complex (PDB 6X6A) (28). In the structure, the ZU5-UPA region from intact NLRP1 (denoted as NLRP1^A^) and UPA portion of the C-terminal fragment of NLRP1 (denoted as NLRP1^B^) bound to a DPP9 molecule are indicated. The schematic of the complex is represented in the right panel.

Human-NLRP1 is an NLR with an atypical domain configuration with PYD, NACHT, LRR, a function to find domain (FIIND), and CARD domains from the N-terminal to the C-terminal side (**Figure 5A**) (67, 68). FIIND is further divided into ZU5 (found in ZO-1 and UNC5) and UPA (found in UNC5, PIDD, and ankyrins) subdomains. Autoproteolysis between these two subdomains is important for NLRP1 activation (69, 70). Gain-of-function mutations in NLRP1 are known to cause inflammatory diseases, particularly in the skin (67, 71). NLRs generally signal through their N-terminal PYD or CARD domains, but previous studies have shown that the C-terminal CARD domain is responsible for signaling in NLRP1 (69). The trigger for the activation of NLRP1 has been unknown for many years. However, recently, it was shown that the activation is triggered by the cleavage of human NLRP1 *via* the enteroviral 3C protease at the linker between PYD and NACHT (Q130-G131) (**Figure 5A**) (72, 73). The resulting N-terminal glycine activates the N-glycine-mediated degradation pathway, which degrades the autoinhibitory NACHT-LRR domain and releases a C-terminal fragment (UPA-CARD) to activate NLRP1 (74–76). The CARD domain of the free C-terminal fragment forms filaments, through which ASC or procaspase-1 is recruited to form the inflammasome (77, 78). Similarly, mouse NLRP1B is cleaved near its N-terminal side by bacterial lethal toxin proteases, resulting in the initiation of N-terminal degradation and release of the C-terminal activating fragment (79–81). In addition, ubiquitination of NLRP1B by bacterial pathogen *Shigella flexneri* IpaH7.8 E3 ubiquitin ligase has shown to activate NLRP1B (75). Dipeptidyl peptidase (DPP)8 and DPP9 are cytoplasmic dipeptidyl peptidases that bind directly to NLRP1 and inhibit its activation. Inhibition of NLRP1 by DPP8/DPP9 is counteracted by DPP8/DPP9 inhibitors; DPP8/DPP9 inhibitors activate NLRP1 (74, 82–85). Furthermore, human NLRP1 has been shown to be activated by recognition of virus-derived double-stranded RNA (dsRNA) (86).

Regarding the structural biology of NLRP1, the structure of the region containing the central NACHT-LRR domain has not yet been elucidated. However, cryo-EM analysis has recently revealed a mechanism by which the C-terminal fragment released by the N-terminal degradation is repressed by DPP9 (**Figure 5B**) (27, 28). A ternary complex consisting of one molecule of full-length NLRP1 (NLRP1^A^) and one molecule of the C-terminal fragment of NLRP1 (NLRP1^B^) with one molecule of DPP9 was elucidated. The complex contained full-length NLRP1A, but only DPP9, the FIIND domain of NLRP1^A^ (ZU5 and UPA), and the UPA portion of NLRP1^B^ were resolved by cryo-EM analysis; other portions were not observed in the cryo-EM map. A peptide of approximately 10 residues on the N-terminal side of NLRP1^B^, generated by the auto-cleavage of the FIIND domain, was inserted into the substrate recognition pocket of DPP9. Thus, inhibitors of DPP9 that bind to this pocket competitively drive out NLRP1^B^, allowing the C-terminal fragment to escape capture by DPP9 and become active. In the complex structure, interactions between ZU5 of NLRP1^A^ and DPP9, UPA of NLRP1^B^ and DPP9, as well as UPAs of NLRP1^A^ and NLRP1^B^ were identified. It has been shown that mutations in the first two parts cause constitutive activation of NLRP1, while mutations in the latter inhibits NLRP1 activation. This suggests that not only the C-terminal fragment of NLRP1^B^, which binds to the substrate recognition pocket of DPP9, but also the ZU5 domain of full-length NLRP1^A^ is important for the inhibition of activation of the C-terminal fragment of NLRP1^B^ by DPP9. In other words, when a small amount of the C-terminal fragment is unintentionally generated, the presence of intact NLRP1 provides a checkpoint to prevent unintended activation of NLRP1 by the DPP9 inhibitory mechanism (27, 28). However, increased production of the C-terminal fragment of NLRP1, such as during viral infection, is thought to decrease intact NLRP1, rendering this DPP9 checkpoint dysfunctional, resulting in the release of the C-terminal fragment, which in turn leads to NLRP1 activation.

However, the mechanism of NLRP1 activation remains unclear. In other NLRs, oligomerization *via* the NACHT-LRR portion causes spatial proximity between the signaling domains, which is thought to trigger activation (17, 18). The NACHT-LRR portion of NLRP1 acts as a domain that inhibits the release of the C-terminal fragment in the functional degradation mechanism (75, 76) described above. Moreover, the NACHT-LRR portion of NLRP1 is involved in dsRNA recognition during NLRP1 activation by a recently reported virus-derived dsRNA (86). Further studies are required to elucidate the precise role of the NACHT-LRR portion of NLRP1.

### NOD2

NOD2 is a member of the NLRC family, and its mutations are closely associated with inflammatory diseases such as Crohn’s disease, Blau syndrome, and early-onset sarcoidosis (87, 88), requiring further functional explanation based on its structural biology. It has two CARD domains on its N-terminal side as signaling domains (**Figure 1A**). NOD2 is believed to be activated by muramyl dipeptide (MDP) from the bacterial cell wall (89, 90). In addition, diverse stimuli, including *Salmonella typhimurium* effector protein SipA and SopE have been identified to activate NOD2 (91, 92). Upon activation, NOD2 oligomerizes to bring its CARD domains into proximity, recruiting downstream RIPK2 through CARD-CARD interaction, and ultimately activating nuclear factor-κB and inducing an inflammatory response (89, 90).

To date, the crystal structure of the ADP-bound, inactivated form of NOD2 lacking the CARD domain has been determined (**Figure 1B**) (29). Similar to the inactivated forms of NLRC4 (16) and NLRP3 (36) (**Figure 1B**), the NACHT domain maintains a closed structure by binding ADP. Mutations that disrupt the interactions between NACHT subdomains increase NOD2 activation, indicating that the interactions between these subdomains are important in maintaining the inactivated conformation (29). The MDP-binding site inferred from previous mutation experiments (93) was located on the concave side of the LRR (29). Mutations in the residues at this site have been shown to decrease the MDP responsiveness of NOD2. It is thought that the binding of MDP to this site induces a conformational change that results in oligomer formation; however, the details have not been yet clarified. Disease-associated mutations are distributed throughout NOD2. Among these, gain-of-function mutations are particularly prevalent at residues located at the interface between the NACHT subdomains. Few studies reported that NOD2 functions by binding to the membrane (94), and some disease-associated mutations are located on positively charged surface residues of HD2, suggesting that NOD2 may bind to the membrane at this site (29).

### NLRP9

NLRP9, a member of the NLRP family, together with DExH box RNA helicase (DHX) 9, recognizes rotavirus RNA in intestinal epithelial cells to form inflammasomes and is involved in resistance to rotavirus infection (95). Recently, the crystal and cryo-EM structures of an ADP-bound inactivated form of NLRP9 lacking the PYD domain have been reported (**Figure 1B**) (30), and both structures are nearly identical. ADP-bound NLRP9, like other inactive forms of NLRs (16, 29, 36), has a closed NACHT domain. Approximately 10 residues on the C-terminal side of NLRP9 have been found to fold back from the tip of LRR to the concave side of LRR, forming an extensive interaction with the concave side of LRR. As discussed, the concave surface of the LRR of each NLR has a distinctive function (23–25, 29, 36, 96). Moreover, it has been speculated that this region of NLRP9 may also play an important role in interactions with other proteins and oligomer formation. However, most mechanisms remain unclear, including the mechanism of inflammasome activation by NLRP9 and the recognition of virus-derived RNA in cooperation with DHX9 (95).

### NLRP6

NLRP6 is a member of the NLRP family and, as with NLRP9, plays an important role in immune responses in intestinal epithelial cells (97, 98). Similar to NLRP9, it cooperates with DHX15, an RNA helicase, to bind to the RNA introduced by enteric viruses and induce interferon production through MAVS (98). It is also known to sense microbiota-associated metabolites and form ASC-dependent inflammasomes (97). For NLRP6, the structures of the PYD domain and its filament structure are known (99). However, the structure of the remaining NACHT-LRR portion remains unclear. Recently, it has become clear that liquid-liquid phase separation (LLPS), which has attracted much attention recently because of its involvement in various biological phenomena, plays an important role in the activation of NLRP6 (100). *In vitro* and intracellular experiments indicate that dsRNA induces LLPS formation of NLRP6 and that this LLPS formation is important for the activation of the NLRP6 inflammasome. The adaptor molecule ASC solidifies the LLPS of NLRP6 and activates the inflammasome. The poly-lysine sequence in the NACHT domain of NLRP6 has been shown to be important for LLPS formation. LLPS-mediated NLRP6 activation is a novel NLR inflammasome activation mechanism, and whether similar mechanisms exist for other NLRs must be further investigated in the future.

## Concluding remark

The past decade has provided a better understanding of the activation mechanisms of NLRs based on structural biology studies. The mechanism of ring-shaped oligomers as a starting point for downstream adaptor signaling, as evidenced structurally in NLRC4 and postulated in NLRP3, is now clear. However, there is little structural evidence regarding the activation mechanism of NLRs. For instance, how ATPase activities of NLR are involved in the activation, how the conformational change leading to the oligomerization is triggered, and further studies are essential to clarify the activation mechanisms of NLRs. In contrast, the mechanism by which the NACHT-LRR portion of NLRP1 is degraded and that by which the released C-terminal fragment serves as a scaffold for downstream adaptor molecules have been elucidated. Moreover, the mechanism by which NLRP6 condensed by LLPS serves as a scaffold for downstream adaptor molecules has also been revealed. Although these activation mechanisms promote recruitment of downstream adaptor molecules by increasing the local concentration of signaling domains, diverse NLR activation mechanisms are still being uncovered. NLRs are involved in a variety of diseases, and their detailed activation mechanisms based on structural biology should be further studied to aid in developing therapeutic agents.

## Author contributions

The author confirms being the sole contributor of this work and has approved it for publication.

## Funding

This work was supported by a Grant-in-Aid from the Japanese Ministry of Education, Culture, Sports, Science, and Technology (Grant Nos. 22H02556 and 19H03164).

## Conflict of interest

The author declares that the research was conducted in the absence of any commercial or financial relationships that could be construed as a potential conflict of interest.

## Publisher’s note

All claims expressed in this article are solely those of the authors and do not necessarily represent those of their affiliated organizations, or those of the publisher, the editors and the reviewers. Any product that may be evaluated in this article, or claim that may be made by its manufacturer, is not guaranteed or endorsed by the publisher.
